# Unilateral optic neuropathy following subdural hematoma: a case report

**DOI:** 10.1186/1752-1947-4-19

**Published:** 2010-01-22

**Authors:** Alexandra Kretz, Christoph Preul, Hans-Joerg Fricke, Otto W Witte, Christoph Terborg

**Affiliations:** 1 Department of Neurology, University of Jena Medical School, Erlanger Allee 101, Jena D-07747, Germany; 2 Department of Internal Medicine, Haemato-Oncology, University of Jena Medical School, Erlanger Allee 101, Jena D-07747, Germany; 3 Department of Neurology, Asklepios Klinik St. Georg, Lohmühlen Str. 5, Hamburg D-20099, Germany

## Abstract

**Introduction:**

Unilateral optic neuropathy is commonly due to a prechiasmatic affliction of the anterior visual pathway, while losses in visual hemifields result from the damage to brain hemispheres. Here we report the unusual case of a patient who suffered from acute optic neuropathy following hemispherical subdural hematoma. Although confirmed up to now only through necropsy studies, our case strongly suggests a local, microcirculatory deficit identified through magnetic resonance imaging *in vivo*.

**Case presentation:**

A 70-year-old Caucasian German who developed a massive left hemispheric subdural hematoma under oral anticoagulation presented with acute, severe visual impairment on his left eye, which was noticed after surgical decompression. Neurologic and ophthalmologic examinations indicated sinistral optic neuropathy with visual acuity reduced nearly to amaurosis. Ocular pathology such as vitreous body hemorrhage, papilledema, and central retinal artery occlusion were excluded. An orbital lesion was ruled out by means of orbital magnetic resonance imaging. However, cerebral diffusion-weighted imaging and T2 maps of magnetic resonance imaging revealed a circumscribed ischemic lesion within the edematous, slightly herniated temporomesial lobe within the immediate vicinity of the affected optic nerve. Thus, the clinical course and morphologic magnetic resonance imaging findings suggest the occurrence of pressure-induced posterior ischemic optic neuropathy due to microcirculatory compromise.

**Conclusion:**

Although lesions of the second cranial nerve following subdural hematoma have been reported individually, their pathogenesis was preferentially proposed from autopsy studies. Here we discuss a dual, pressure-induced and secondarily ischemic pathomechanism on the base of *in vivo *magnetic resonance imaging diagnostics which may remain unconsidered by computed tomography.

## Introduction

Unilateral optic neuropathy (ON) following subdural hematoma has been confirmed by necropsy studies. In these studies, microcirculatory compromise of the optic nerve was proven as a pathogenic mechanism [1]. In this case report, diffusion-weighted images (DWI) of magnetic resonance imaging (MRI) scans showed signal alteration in the ipsilateral optic nerve as a strong evidence for the development of microvascular deficit. Thus, our case is in line with results from autopsies. To the best of our knowledge, this is the first case presentation that demonstrates microvascular impairment with optic neuropathy *in vivo*.

## Case presentation

**Figure 1:**
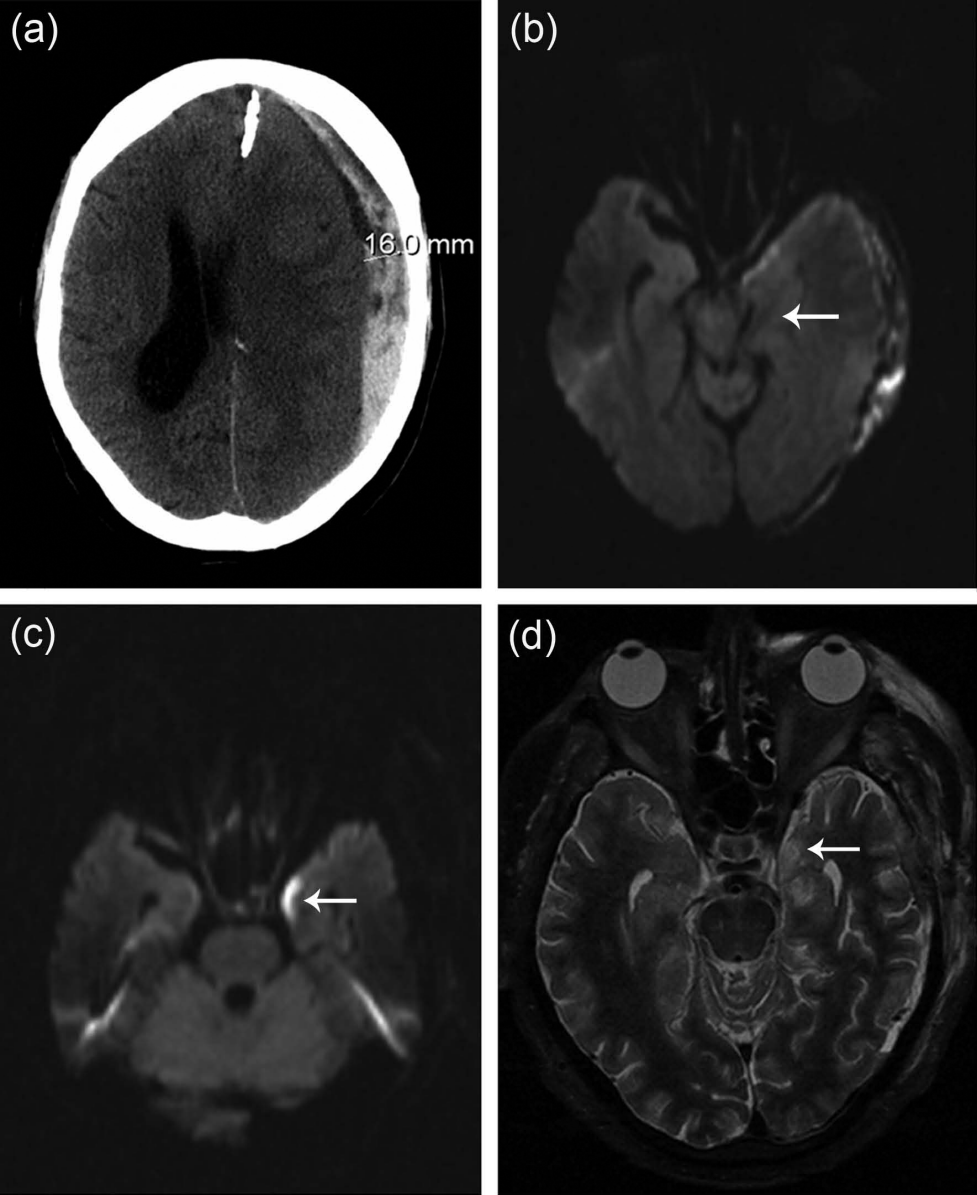
**Computed tomography of subdural hematoma and cerebral magnetic resonance imaging depicting early uncal herniation and mesiotemporal ischemic brain injury**. **(A) **Preoperative cranial computed tomography scan reveals space-occupying subdural hematoma covering the left convexity. The hematoma causes a considerable midline shift at the level of the lateral ventricles. **(B) **Early diffusion- weighted magnetic resonance imaging (diffusion-weighted imaging) depicts a slight uncal herniation and a midline deviation at the level of the chiasmatic basal cistern. **(C) **Diffusion-weighted imaging sequences four days after surgery show circumscribed signal hyperintensity in the left uncal region indicative of local ischemic injury. Note the close anatomic proximity to the distal prechiasmatic course of the left optic nerve. **(D) **Corresponding hyperintensity in T2-weighted images indicates edema of the left temporomesial lobe that is consecutive to hypoperfusion and residual to preceding uncal herniation.

A 70-year-old Caucasian man of German nationality receiving warfarin therapy for the primary prevention of chronic atrial fibrillation was admitted to our hospital due to symptoms of a coronary syndrome. An initial international normalized ratio (INR) of 1.7 was elevated to therapeutic ranges (INR = 2.5). Three days later, the patient was found comatose after a first-ever generalized seizure. Cerebral computed tomography (CT) revealed a subdural hematoma measuring 16 mm at its maximum thickness and covering almost the entire left convexity which caused a massive midline shift (Figure 1A). Rapid cerebral MRI of the same day depicted a beginning ipsilateral uncal herniation towards the chiasmatic cistern (Figure 1B). Immediately after INR normalisation, surgical evacuation of the subdural hematoma and decompressive craniectomy were performed without any complications.

Two days after extubation and recovery from anaesthesia, the patient complained of severe sinistral visual loss. Neurologic and ophthalmologic examinations confirmed a severely reduced visual acuity on his left eye with concomitant afferent pupillary defect. A normal vascular fundoscopy and the lack of papilledema led to the working diagnosis of posterior ON. Four days after the surgery, a follow-up DWI of the cerebral MRI (Figure 1C) showed a signal hyperintense lesion within the left lateral, mesiobasal temporal lobe that was immediately adjacent to the distal prechiasmatic course of the affected optic nerve. Dull signal attenuation in corresponding apparent diffusion coefficient (ADC) maps, and gadolinium enhancement in T1 (not shown) led to the diagnosis of a subacute cerebral ischemic event.

The corresponding hyperintensity of T2-weighted images indicated focal brain edema in line with regional hypoperfusion and the preceding uncal shift (Figure 1D). Thus, pathomorphologic and sequence-specific MRI criteria suggest a primary mechanic compression followed by secondary microcirculatory impairment of the afflicted brain area. In support of this hypothesis, DWI alterations were not manifest at the initial MRI diagnostics. Although not directly proven by MRI but due to its close anatomic course and the temporal coincidence of optic nerve affliction, an equal dual pathomechanism was believed to be the cause of the appearance of ON. Further cerebral or orbital pathologies were not apparent (not shown). Within weeks, the patient was released for rehabilitation without further visual improvement.

## Discussion

To date, only a few cases of ON following subdural hematoma are presented, and their pathomorphologic *in vivo *findings do not at all elucidate its aetiology. Generally, reports do not present the existence of papilledema or of radiologic *in vivo *evidence of optic nerve compression by mass effects [2]. In contrast, autopsy studies confirm optic nerve necrosis that is remote from space occupying lesions [1].

The pathophysiology of ON has been discussed in the context of diverse aetiological events, and adequate diagnostic approaches have already been proposed. Table 1 provides an overview of selected publications focussing on key diagnostic means to identify and characterize vascular, mechanical, and pharmacologic aetiologies of ON (Table 1).

**Table 1:** Overview of the different aetiologies of optic neuropathy.

Optic Neuropathy	Pathophysiology	Diagnostic Means	Diagnostic Parameter	**Ref**.
**AION (anterior ischemic optic neuropathy)**	Infarction of the ONH due to perfusion deficit of the SPCA	Infrared pupillography	Latent period of the pupil light reflex	[6]
		Goldmann perimetry	Visual field impairment, (cecocentral) scotoma	[7]
		VEP	Retarded P100 latency, diminished potential amplitude	[8]
		Color fundus photography	Crowded, edematous disc, peripapillary hemorrhage	[7]
		Fluorescein angiography (i.v.)	Vascular morphology and topology; discal perfusion delay	[7,8]
		Ocular blood flow system	Pulse synchronic alterations of intraocular pressure	[9]
		
		Optical coherence tomography, e.g. by HRT	Circumferential scan of the ONH for quantitative, morphological analysis, e.g. cup size/volume, RNFL thickness	[10]
		Scanning laser polarimetry, by confocal scanning laser ophthalmoscope	Estimation of RNFL thickness by retardation of polarized scanning laser beam	[11]
		Laser doppler flowmeter, e.g. by HRF	Capillary blood flow of retina and choroidea via transpupillary laser scanning ophthalmoscopy	[12]
		(Color) doppler imaging	Blood flow of intraorbital, retroorbital, small choroideal vessels including SPCA	[13]
		MRI	DWI restriction, reduced ADC map	[4]
		Fundoscopy	Edematous disc, macular infarction, subsequent disc pallor	[3,4]

**PION (posterior ischemic optic neuropathy)**	Retrolaminar ischemia due to hypo-perfusion of the Zinn-Haller circle (pial, choroideal vessels, PCA)	Fundoscopy	Initially normal disc, pallid after weeks	[14]
		MRI		[14]

**CRAO (central retinal artery occlusion)**	Perfusion deficit of the central retinal artery	Clinical examination fundoscopy	Absent papillary reflex or RAPD, cherry red spot of the macula	[15]

**SAH (subarachnoid hemorrhage due to intracranial aneurysma rupture)**	Perfusion deficit during surgical procedure (e.g. clipping), emboli, vasospasm	Cerebral angiography	Vessel calibres: aneurysm, emboli, vasospasm	[16]

**CST (cavernous sinus thrombophlebitis)**	Venous infarction due to thrombosis of ophthalmic veins	Venography	Absence of contrast filling in orbital veins	[5]
		MRI	DWI restriction, ADC reduction along optic nerve	[5]

**Compressive optic neuropathy**	Mucus in paranasal sinus	Orbital CT	Erosion of optic canals	[17]

**Toxic**	e.g. IFNα therapy	Fundoscopy	Edematous disc	[18]

Pathophysiological concepts and key diagnostics as well as diagnostic parameters are provided together with selected references from the literature.

ADC, attenuated diffusion coefficient; AION, anterior ischemic optic neuropathy; CRAO, central retinal artery occlusion; CST, cavernous sinus thrombophlebitis; CT, computed tomography; DWI, diffusion weighted imaging; HRF, Heidelberg-Retina-Flowmeter; HRT, Heidelberg-Retina-Tomogram; MRI, magnetic resonance imaging; ONH, optic nerve head; PION, posterior ischemic optic neuropathy; PCA, posterior ciliary arteries; RAPD, relative afferent pupillary defect; RNFL, retinal nerve fibre layer; SAH, subarachnoid hemorrhage; SPCA, short posterior ciliary artery; VEP, visual evoked potential.

At present, only a few cases refer to the diagnostic value of MRI in ON. By means of MRI, unilateral [3] and simultaneous bilateral [4] ischemic ON were diagnosed. It was concluded that DWI and ADC maps of MRI may be useful in detecting ischemia of any white matter tracts that are disparate from the brain and spinal cord. MRI diagnostics may even prove suitable in distinguishing ischemic events from optic neuritis [3], while also providing the opportunity to simultaneously detect anterior ischemic optic neuropathy (AION) and posterior ischemic optic neuropathy (PION). Likewise, MRI venography in relation to DWI and ADC maps unequivocally confirmed that ON can be caused by cavernous sinus thrombophlebitis [5].

Using the MRI technique, we now provide a pathophysiologic insight on ON following space occupying subdural hematoma early *in vivo*. Radiological signs of herniation were discrete, although highly sensitive MRI revealed a mechanic, pressure-induced brain lesion of the mesiobasal temporal lobe in proximity to the affected optic nerve. However, focal ischemic injury was missed in detecting basal brain shift by CT. Since MRI pathologies fulfilled the criteria of ischemic compromise, we suggest that the local increase in intracranial pressure (ICP) exceeded the perfusion pressure of both structures, namely the formation of the uncus and the nearby passing optic nerve. Consistent with this notion of a microcirculatory deficit, the lesion did not follow the characteristic extent of a vascular territory.

## Conclusions

We suggest that rare cases of acute ON following subdural hematoma are due to local pressure-induced optic nerve infarction. This pathomechanism may remain neglected when massive brain shift is lacking or when CT is the only diagnostic means. The use of serial MRI may help balance the discrepancy between the paucity of clinical reports and frequent neuropathological findings of anterior visual pathway damage in space-occupying brain injury [1].

## Abbreviations

ADC: apparent diffusion coefficient; AION: anterior ischemic optic neuropathy; CT: computed tomography; DWI: diffusion-weighted imaging; FLAIR: fluid attenuated inverse recovery; ICP: intracranial pressure; INR: international normalized ratio; MRI: magnetic resonance imaging; ON: optic neuropathy; PION: posterior ischemic optic neuropathy.

## Consent

Written informed consent was obtained from the patient for publication of this case report and any accompanying images. A copy of the written consent is available for review by the Editor-in-Chief of this journal.

## Competing interests

The authors declare that they have no competing interests.

## Authors' contributions

AK and CP interpreted the patient data and clinical course regarding the neurological disease. HF, OW and CT were major contributors in conceiving and writing the manuscript. All authors read and approved the final manuscript.
